# The Cultural Divide: Exponential Growth in Classical 2D and Metabolic Equilibrium in 3D Environments

**DOI:** 10.1371/journal.pone.0106973

**Published:** 2014-09-15

**Authors:** Krzysztof Wrzesinski, Adelina Rogowska-Wrzesinska, Rattiyaporn Kanlaya, Kamil Borkowski, Veit Schwämmle, Jie Dai, Kira Eyd Joensen, Katarzyna Wojdyla, Vasco Botelho Carvalho, Stephen J. Fey

**Affiliations:** 1 Tissue Culture Engineering Laboratory, Department of Biochemistry and Molecular Biology, University of Southern Denmark, Odense, Denmark; 2 Protein Research Group, Department of Biochemistry and Molecular Biology, University of Southern Denmark, Odense, Denmark; 3 Department of Biology, University of Copenhagen, Copenhagen, Denmark; University College London, United Kingdom

## Abstract

**Introduction:**

Cellular metabolism can be considered to have two extremes: one is characterized by exponential growth (in 2D cultures) and the other by a dynamic equilibrium (in 3D cultures). We have analyzed the proteome and cellular architecture at these two extremes and found that they are dramatically different.

**Results:**

Structurally, actin organization is changed, microtubules are increased and keratins 8 and 18 decreased. Metabolically, glycolysis, fatty acid metabolism and the pentose phosphate shunt are increased while TCA cycle and oxidative phosphorylation is unchanged. Enzymes involved in cholesterol and urea synthesis are increased consistent with the attainment of cholesterol and urea production rates seen in vivo. DNA repair enzymes are increased even though cells are predominantly in G_o_. Transport around the cell – along the microtubules, through the nuclear pore and in various types of vesicles has been prioritized. There are numerous coherent changes in transcription, splicing, translation, protein folding and degradation. The amount of individual proteins within complexes is shown to be highly coordinated. Typically subunits which initiate a particular function are present in increased amounts compared to other subunits of the same complex.

**Summary:**

We have previously demonstrated that cells at dynamic equilibrium can match the physiological performance of cells in tissues in vivo. Here we describe the multitude of protein changes necessary to achieve this performance.

## Introduction

There are two extreme conditions for cell growth. The first is characterized by exponential growth of cultures using classical 2D culture techniques typically involving a cycle of trypsinisation, inoculation, growth and re-trypsinisation and a short doubling time (usually a few days). The second is characterized by cells which have reached a dynamic equilibrium, growing either in 3D tissues or tissue-like conglomerates, in which the doubling time is long (a month or longer) and in which trypsin is rarely, if ever, used.

We have used a cell culture system based on the maintenance of cell clusters in suspension to induce cells to reach the dynamic equilibrium. In this system a culture chamber is rotated on a clinostat in a standard cell culture incubator (also known as microgravity cultures). The cell clusters are initiated by centrifuging cells into inverted pyramidal indentations in a special culture plate. After incubation these clusters are released into the rotating culture chamber and then treated in essentially the same way as one would treat cells in a culture flask. During culture, these clusters round up and are therefore termed 3D microgravity ‘spheroids’ in this manuscript. Previously, we have shown that HepG2/C3A cells need 18 days to complete the transition from exponential growth to dynamic equilibrium [1]. Once recovered, spheroids exhibit a metabolism that is stable from 18 to at least 42 days in culture and produce urea, cholesterol and ATP at physiological levels (i.e. those seen in the human body) [2]. Rotation of the culture chamber creates a flow of media past the spheroids which in turn allows them to grow to sizes about ten times larger in diameter than spheroids in static, hanging drop technologies. These microgravity spheroids are more predictive of human toxicology than classically cultured immortal or primary human hepatocytes (at least for the 5 drugs tested) [3].

This is a general phenomenon. A physiological behavior closer to that seen *in* *vivo* has been described in many other cell and stem cell lines once they have been cultured for approximately 18 days in appropriate environments. These include human Caco-2, HT29 SW480 and SW1222 (used for modelling intestinal compound or drug transport [4], [5] and [6] and [7]); HT29 cells (for lipid raft assembly [8]); MDCK cells (to model polarised epithelial structure of the kidney, lung and breast [9]); MCF-10A spheroids (to mimic growth-arrested breast acini [10]); primary hepatocytes (which regain their ultrastructure [11] and some functionality [12]); differentiated Huh-7 (which can be productively infected with hepatitis C virus [13]); C2C12 myoblasts (form syncytia and express skeletal muscle specific markers [14]). Stem cells also need similar periods of trypsin-free time in culture to differentiate. Stem cells can form cortical-like tissues [15]) dopaminergic NT2N neurons [16], inner ear sensory epithelia [17] and transplantable photoreceptor cells [18].

Since most of this gain in physiological attributes occurs spontaneously, we hypothesize that these observations may in part be a reflection of cells reaching their dynamic equilibrium. A proteomic comparison of an adenocarcinoma with normal colon tissue, have shown that the changes seen are comparable to those seen when exponentially growing Caco-2 cells are compared with those cultured for 18 days [19]. This underlines the similarity between immortal cells at dynamic equilibrium with normal tissue. All the data presented here stem from work with the HepG2/C3A cell line.

The aim of this study was therefore to determine what changes occur in the proteome as the cells recover from exponential growth and reach dynamic equilibrium. This is of critical importance since the cells at dynamic equilibrium exhibit a metabolism close to the physiological performance of the cells in the intact organism. As far as we can ascertain, this is the first time that this has been done. The results show that cells grown as spheroids in 3D culture are radically different (both structurally and metabolically) to cells grown in classical 2D cell culture. These differences correlate well with the observations that 3D hepatocyte spheroids can mimic the physiological responses seen by the human liver *in* *vivo*.

## Materials and Methods

### Cell culture (2D culture)

The methods for growing the hepatocellular carcinoma cell line, HepG2/C3A (ATCC CRL-10741) using either classical cell culture methods or as 3D spheroids are given in detail in [2]. Briefly, cells were grown in standard tissue culture conditions (87.5% D-MEM (containing 1 g glucose/L, (Gibco Cat. no. 31885-023), 1% Non-Essential Amino Acids (Gibco Cat. no. 11140-035), 10% FCS (Foetal calf serum, Sigma Cat. no. F 7524), 0.5% Penicillin/Streptomycin (Gibco Cat. no. 15140-122), 1% GlutaMAX (Gibco Cat. no. 35050-038), 37°C, 5% CO_2_ 95% air). When necessary they were trypsinised for 3 min using 0.05% Trypsin/EDTA (Gibco Cat. no. 15400-054), diluted 1∶4 and sown out into falcon flasks or microtitre plates.

### Spheroid culture conditions (3D culture)

The HepG2/C3A cell spheroids were prepared using AggreWell 400 plates (Stemcel Technologies Cat. no. 27845). 1.2×10^6^ cells were added to each well, centrifuged for 3 min at 100×g and then left overnight to form spheroids.

### Spheroid culture in bioreactors

The bioreactor (MC2 Biotek, Hørsholm Cat. no. 010) humidity chamber was filled with distilled sterile water, and the growth chamber was prewetted with growth medium for 24 h before use. During this time they were rotated in an incubator. Spheroids were detached from the AggreWell plates, their quality checked by microscopy and then placed into open bioreactors. The bioreactors were closed and the spheroids cultivated at 37°C, 5% CO_2_ 95% air in a humidified incubator for a minimum of 21 days, exchanging the medium every two to three days. Before samples were analysed by other assays, the spheroid batch quality was assessed by staining for 3 min. with 0.4% Tryphan Blue (Gibco Cat. no. 15250-061) and accepted if they showed greater than 90% viability.

### Protein determination in spheroids

The amount of protein present in a sample was determined either by using the fluorescence based ProStain Protein Quantification Kit (Active Motif Inc. Cat. no. 15001) or estimated from the shadow area of the spheroids as described previously [3].

### DNA determination

The DNA Quantitation Kit (Sigma-Aldrich Cat. no. DNAQF) was used to determine the amount of DNA present in the experimental samples. The assay is based on fluorescent dye, bisBenzimide H 33258 (Hoechst 33258), which binds primarily to AT sequences in the minor groove of double-stranded DNA (dsDNA). The assay was set up in a 96-well plate format and the readouts were measured using a multi-mode microplate reader FLUOStar Omega (BMG Labtech, Ortenberg-Germany). Fluorescence excitation and emission wavelengths were set to 355 nm and 460 nm respectively. Average results from 5 cycles with 10 individual measurements per cycle have been used to calculate the DNA content of the sample.

### Sample preparation for mass spectrometry

Samples from classical 2D cell culture were collected 5 days after trypsinisation and samples from 3D spheroid culture were collected 21 days after spheroid culture initiation. Collected samples were washed 7 times with warm (37°C) Hanks’ Balanced Salt Solution (HBSS without Ca^++^ and Mg^++^, Gibco Cat. no. 14175-053). After removal of all the remaining HBSS the samples were snap frozen in liquid nitrogen and stored until further processing.

The HepG2/C3A cells were lysed in 400 µL of lysis buffer (4%(w/v) SDS, 100 mM Tris/HCl pH 7.6 0.1 M DTT) containing both a protease inhibitor cocktail (Complete Mini, Roche) and a phosphatase inhibitor (PhosphoSTOP, Roche). The lysate was heated at 95°C for 5 min and then sonicated 3 times for 10 sec at 10 W output. Cell debris was removed by centrifugation at 16,000×g for 5 min at 4°C. The clarified supernatant was transferred into a fresh tube and kept at −80°C until use. The protein concentration was determined by amino acid analysis.

### Proteolytic digestion

Proteins were digested into peptides according to the ‘filter-aided sample preparation’ (FASP) method [20]. Briefly, 100 µg of the cell lysate was mixed with 200 µL of ‘urea solution’ (8 M urea in 0.1 M Tris/HCl, pH 8.5) in the filter unit (Nanosep 10K Omega, PALL) and centrifuged at 14,000×g for 15 min. Two further 200 µL aliquots of urea solution were added and centrifuged at the same speed. The proteins were alkylated in the dark with 100 µL of IAA solution (0.05 M iodoacetamide in urea solution) for 20 min on the thermo-mixer at 600 rpm and then centrifuged at 14,000×g for 10 min. Subsequently, the filter unit was washed with 100 µL of urea buffer and centrifuged at 14,000×g for 10 min. This step was repeated twice to ensure complete removal of the IAA solution. Subsequently, the urea buffer was exchanged by 50 mM NH_4_HCO_3_ buffer by three consecutive on-filter washes and centrifugations (14,000×g for 10 min). The proteins were digested with trypsin (enzyme to protein ration 1∶50 w:w) at 37°C for 18 h. The peptide solution was collected by transferring the filter unit to a new collection tube and centrifugation at 14,000×g for 10 min. Peptide elution was repeated again by adding 40 µL 50 mM NH_4_HCO_3_ buffer and centrifugation at 14,000×g for 10 min. The peptide solution was acidified with TFA to a final concentration of 0.5% and dried under vacuum.

### Stable isotope dimethyl labelling

On column stable isotope dimethyl labelling was performed by using in-house made C18 microcolumns. The microcolumns were washed twice with 100 µL acetonitrile (ACN) and equilibrated twice with 100 µL 1% trifluoroacetic acid (TFA). Tryptic peptides were dissolved in 100 µL 1% TFA and applied to the microcolumns, followed by two 100 µL washes in 0.1% TFA. The labelling reagent was prepared in 50 mM sodium phosphate buffer pH 7.5 and mixed with of 50 µL of 4% (v/v) of either CH_2_O (light) or CD_2_O (heavy) and 250 µL of 0.6 M sodium cyanoborohydride (NaBH_3_CN). The microcolumns were flushed five times with 100 µL labelling reagent individually (leaving the column to stand for about 10 min each time) and were washed twice with 100 µL 0.1% TFA. The labelled peptides were eluted with 100 µL of 50% ACN in 0.1% TFA and then with 100 µL 70% ACN in 0.1% TFA into the same tube. Equal amounts of labelled peptides from light and heavy labels were mixed together and dried under vacuum. The peptide solutions were dissolved in 100% formic acid (FA) and purified using microcolumns containing a C8 plug. After drying under vacuum, the peptides were dissolved in 0.1% FA and analysed by LC-MS/MS.

### LC-MS/MS analysis and data processing

Peptide mixtures were analyzed by nanoflow liquid chromatography using the EASY-nLC system (Thermo Fisher Scientific, Bremen, Germany). The 15 cm analytical column consisted of a 100 µm internal diameter capillary packed with reverse-phase C18-AQ, (3 µm, Dr. Maisch GmbH, Ammerbuch, Germany). A two mobile phase system consisting of buffer A (0.1% FA) and buffer B (95% ACN, 0.1% FA) was used. The gradient consisted of 164 min linear gradient from 100–65%/0–35% buffer A/B at a flow rate of 200 nl/min, followed by a 10 min gradient from 65–5%/35–95% buffer A/B at a flow rate of 200 nl/min, and a 6 min wash using 100% buffer A using the same flow rate. The eluted peptides were directly electrosprayed into the LTQ Orbitrap Velos (Thermo Fisher Scientific), which was operated in a HCD top 10 mode under the control of the Xcalibur software. The cycle of one full scan was performed at resolution of 30,000 (M/z 350–1800) followed by 10 data dependent scans at resolution of 7,500. The normalized collision energy was 47% and the activation time was 10 ms for acquiring mass spectra. The resulting files were analysed by Proteome Discoverer v.1.3 (Thermo Fisher Scientific). The workflow included peptide identification and quantification. The MASCOT software algorithm was used to search against the 20,317 entries in the human SwissProt Version 2.3 FASTA database. The following parameters were used: trypsin as the proteolytic enzyme, a maximum of two missed cleavage sites were allowed; precursor mass tolerance and fragment mass tolerance was set as 50 ppm and 0.05 Da respectively. Carbamidomethyl of cysteine was set as a fixed modification. Oxidation of methionine, dimethyl (lusine), dimethyl (N-term), dimethyl:2H(4) (lysine), dimethyl:2H(4) (N-term) were chosen as dynamic modifications. The target false discovery rate (FDR) was set as 0.01 by performing a concatenated decoy database search. Precursor ions quantifier processing node was used for quantification of peptides and proteins.

### Indirect immunofluorescence

Samples from 2D cell culture 5 days after trypsinisation and 3D spheroid culture 21 days after spheroid culture initiation were washed four times with warm (37°C) HBSS. They were then fixed for 30 minutes in 4% formaldehyde solution at 4°C. After fixation, the formalin solution was replaced with HBSS for the 2D cell culture and the specimens and stored at −80°C until further processing. For the 3D spheroids, the formalin solution was replaced with the Tissue-Tek O.C.T. compound (Sakura Cat. no. 4583) and the samples were snap frozen in liquid nitrogen. Prior to immunostaining, the samples were warmed to −30°C. 16 µm thick sections of frozen Tissue-Tek O.C.T. embedded spheroid samples were cut using a Microm Kryostat (model: Cryo-Star HM650M). Prepared sections were stored at −80°C until further processing.

The same immunostaining protocol was applied to fixed cells from both 2D and 3D cell culture. Briefly, fixed cells and fixed spheroid sections on glass slides were washed for 15 min with 0.1 M glycine pH 7.4 (buffered with tris-base). Cells were permeabilised with 0.2% triton X-100 in PBS for 5 min and unspecific protein binding sites were blocked by washing for 30 min with 5% BSA (in PBS). Afterwards, the primary antibody, or phalloidin (diluted in 1% BSA in PBS for antibodies, or in PBS for phalloidin) was applied overnight. Next day, the cells were washed with PBS and the secondary antibody was applied for an hour, before being counter-stained with DAPI (diluted in PBS) (Invitrogen D21490) for 5 min. Excess DAPI was washed away with PBS. 0.17 mm thick coverslips were mounted on top of the cells using fluorescence-free mounting medium (Dako 2011-03).

The following primary antibodies were used: Anti-actin (Santa Cruz sc1616), anti-keratin 8 (Thermo Fisher Scientific MA5-15460), anti-acetylated tubulin (Sigma T6793), and anti-alpha tubulin (Sigma T5168). Phalloidin (Molecular Probes C7466) was used to visualise polymerised actin. Alexa fluor 488 conjugated anti-rabbit IgG antibody (Invitrogen A1108) and alexa fluor 555 conjugated anti-mouse IgG antibody (Invitrogen A21422) were used as the secondary antibodies.

Fluorescent images were taken using Leica SP5-X confocal microscope and analysed using Leica LAS AF LITE software.

### Western blot analysis

Cells were lysed by sonication in 20 mM TEA, 4% SDS, 1 mM EDTA buffer. Samples were separated in a Bolt 4–12% Bis-Tris Plus gel using Bolt MES SDS Running Buffer (Invitrogen) following the manufacturer’s instructions. Separated proteins were electro-transferred onto an Immobilon-P Membrane, PVDF (Merck Millipore). Primary antibody binding was detected by incubation with a peroxidase-conjugated secondary antibody and chemiluminescent substrate Luminata Forte (Merck Millipore). Carbonylated proteins were detected and analyzed following derivatization of protein carbonyl groups with 2,4-dinitrophenylhydrazine, using the OxyBlot kit (Merck Millipore). Immunodetection was performed utilizing 10 µg of protein per lane with a primary antibody directed against dinitrophenylhydrazone. Equal loading was demonstrated by using the same amount of each of the samples, separated by SDS PAGE in the same conditions and stained with sensitive Coomassie Blue stain [21]. Density analysis was performed using Image Studio Light (Li-Cor). The optical density for each lane was normalised to the averaged total density of all lanes on the gel and expressed as percentage of the optical density.

### Statistical analysis

The peptide ratios (Heavy/Light) were log2-transformed and normalized by their median for each technical replicate. The peptide ratios were averaged from 4 biological replicates. For multiple measurements of the same peptide within the same biological replicate, we took the mean. This procedure ensured that all peptides of a protein have the same weight when averaging the log2-ratios.

With only 4 biological replicates and large amounts of missing values, advanced statistical tools are required for confident detection of significantly changing proteins. We applied the moderated Bayes test [22] and a modified version of rank products [23]; [24] according to [25]. Significantly changing proteins were defined using a false discovery rate of 5% (p-values corrected for multiple testing according to Storey [26].

For other statistical analysis Student’s t-test or correlation coefficient included in the Excel program were used. For the correlation of the ratio of protein:DNA ratios a two sample equal variance was used and for all other t-tests two sample/unequal variance was used.

### Protein function analysis

Data was analysed with reference to multiple programs and information sources including MedLine, SwissProt, Kegg, Ingenuity and Go protein annotations.

### What is a significant change?

The main task with the data output from modern proteomics experiments is to identify the proteins that are present in different amounts under two experimental conditions. This task is complicated by the noisiness of the data and the large number of proteins that are examined simultaneously. In this manuscript we have used a combination of Limma test, Rank Products, and in some cases a non-parametric Student’s t-test.

In addition we have tested the quality of our data using the heteromer score [27]. Many proteins are found in ‘heteromers’ i.e. where the subunit proteins are known to exist in defined stoichiometries, (e.g. heterodimer, heterotrimer, heterotetramer, etc). A list of all of such proteins was downloaded from the UniProt database (UniProt release 2013_09 - Sep 18, 2013) and used to select proteins from the basic data file (Please see: File S1, tab P1_data). The fold change seen for one subunit of the heteromer was plotted against the fold change of the other and the correlation coefficient (using the least squares correlation available in Excel 2010) corresponds to the heteromer score. This showed that there is a high degree of correlation (R^2^ = 0.933) validating the quality of the data set.

The standard error of the estimate of the heteromer data set, using the 2 sigma level (∼95% probability) demonstrated that a change in the ratio of a single protein by >±1.66 can be considered significant [27].

In the following text, the observed ratios for some groups of proteins have been averaged to give an impression of the abundance of the structure in question or the activity of the pathway. Changes in a group of proteins can be considered significant at a ratio of change lower than that of a single protein (>±1.66). Since the actual value that can be considered significant will depend on the number of proteins involved and the range in their standard deviations, we have focussed on the trends seen for different groups and not used a statistical significance ‘cut-off’. The grouping of the proteins and their actual ratios can be found in File S1 in the series of tabs beginning at P3.

## Results

**Figure 1:**
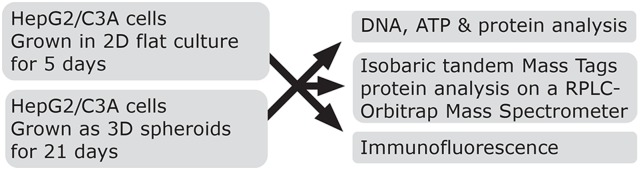
Experimental plan. HepG2/C3A cells were grown using either classical 2D flat culture techniques or as 3D microgravity spheroid techniques and analysed by mass spectrometry, immunofluorescence or using standard assays for DNA, ATP or protein.

The aim of this study was to compare the proteome of HepG2/C3A cells grown in exponential growth (2D cell culture for 5 days) and at dynamic equilibrium (3D spheroid culture for 21 days) to determine whether differences in protein expression can be correlated with the changes in physiological attributes (as described in our previous publications). The proteomes were compared using di-methyl labelling of peptides followed by their separation using nano-liquid chromatography before their analysis by tandem mass spectrometry (figure 1). The cellular content of DNA, protein and ATP was determined and parallel samples were prepared for microscopy and subjected to indirect immunofluorescence using antibodies against actin, tubulin and keratins to substantiate the data obtained.

The basic data used for this analysis (including their statistical analysis) are presented in File S1, tab P1_data. Because of the high number of changes observed and the necessity to describe them in context, only a summary of the data interpretation is given here in the results section. Table 1 provides brief interpretations of the consequences that these changes in protein levels might have. A full description of the results at the level of the individual proteins and their grouping into cellular functions is given in File S1 (tabs P1_data and P3). The section P3 is divided up into individual tabs corresponding to the same subheadings as in text below. Each of these provide additional information and additional references.

**Table 1:** Summary of changes observed when 3D spheroid culture is compared to 2D flat culture.

Protein Group	Change in 3D spheroids
Actin filaments	Increased actin binding proteins and rearranged cytoskeleton
Microtubules	Increased microtubular transport along a rearranged and extended microtubule network
Intermediate filaments	Reduced and rearranged intermediate filament network
Extracellular matrix (ECM)	Reduced ECM (cell surface to volume ratio is decreased)
Glucose and pentose	Increased glucose metabolism (needed to fuel secondary pathways)
metabolism, pyruvate dehydrogenase andTCA cycle	Unchanged TCA cycle
Oxidative phosphorylation andATP synthesis	Reduced Complex IV and ATP synthase (cells in 3D have reached physiological ATP levels)
Fatty acid metabolism	Increased synthesis and reduced oxidation
Cholesterol	Increased synthesis (cells in 3D have reached physiological levels)
Urea production	Increased synthesis (via alternate pathway) (cells in 3D have reached physiological levels)
Oxygen levels	Oxygen transport through spheroid effected by increased non-erythrocyte haemoglobin
	Reduced protein oxidation damage
Cell Growth	High protein:DNA ratio
	Cells in 3D have very low growth rate
The nucleus, DNA repairand packing	DNA organisation and metabolism is rearranged
	No signs of apoptosis or necrosis
Oncogenes and transcriptionfactors	Increase in specific oncogenes and transcription factors and decrease in others (switching)
Transcription	Switching in gene selection
RNA processing	Stabilisation of short-lived mRNAs
hnRNP	Stabilisation of RNA during transport and transcription
Spliceosome	Reduced splicing
	Increased stabilisation of single stranded RNA and circularization
tRNA charging	Increased tRNA synthetases needed for increased transcription and MSC (multi-tRNA synthetase complex) directed homeostasis
Translation	Increased translational activity
	Increased policing to ensure correct transcriptional initiation and accurate reading
The ribosome	Reduced amounts of the large subunits in the cytoplasm and mitochondria
Protein folding	Increased protein folding in the cytoplasm and nucleus mediated by HSP70’s, 90’s, STIP and PPIases
	Increased HSP 27 and 40 directed folding
	Unchanged folding by HSP 60/10 and the TCP-1 Ring complex
Transport	Increased nuclear and microtubular transport
	Increased vesicle sorting and transport in Golgi complex and from *cis* end of Golgi back to rough endoplasmic reticulum (ER) (COPI)
	Unchanged clathrin and COPII (from ER to golgi) vesicle transport
	Reduced endosomal transport from plasma membrane or golgi to lysosome
Ubiquitination and protein	Increased proteasome activity
degradation	Switching of deubiquitinating enzymes
**Conclusion**	**Cells grown in 3D modulate the level of numerous proteins in order to establish an efficient infrastructure and can maintain a physiological performance similar to that seen ** ***in*** ***vivo***

HepG2/C3A cells were grown using classical cell culture techniques (2D) or as spheroids in a MC2 Biotek microgravity rotating bioreactor (3D). Comparative protein levels in these two conditions were determined by mass spectrometric analysis of isotope dimethyl labelled proteins. The data is described in the text and full documentation (including extra references) is provided in the supplementary materials.

The data is given as gene name together with the observed numerical ratio of the amount of the protein in spheroid culture compared to that seen in exponential cultures. The reciprocal has been taken of all values below 1 indicated with a negative sign. In this way, equally significant changes (for example a doubling or a halving of the amount of protein) would be indicated by ‘2.00’ or ‘−2.00’ respectively. Any numerical average values given in the text are the ratio value of the average of the log2 ratios obtained from the study (carried out as biological quadruplicates). Plotting the degree of change against the number of proteins exhibiting that change illustrates that there were more proteins that showed small positive fold changes balanced by a few proteins which displayed larger negative fold changes (File S1, figure in tab P2_Histogram).

### Cellular architecture

Previously we had seen significant changes in the cellular architecture during the formation of spheroids. This included the formation of tight junctions and the partitioning of the plasma membrane into ‘sinusoidal’ and ‘interhepatocyte’ regions. Microvillae were seen on sinusoidal regions and in bile canaliculi-like channels [1].

### Actin filaments

**Figure 2:**
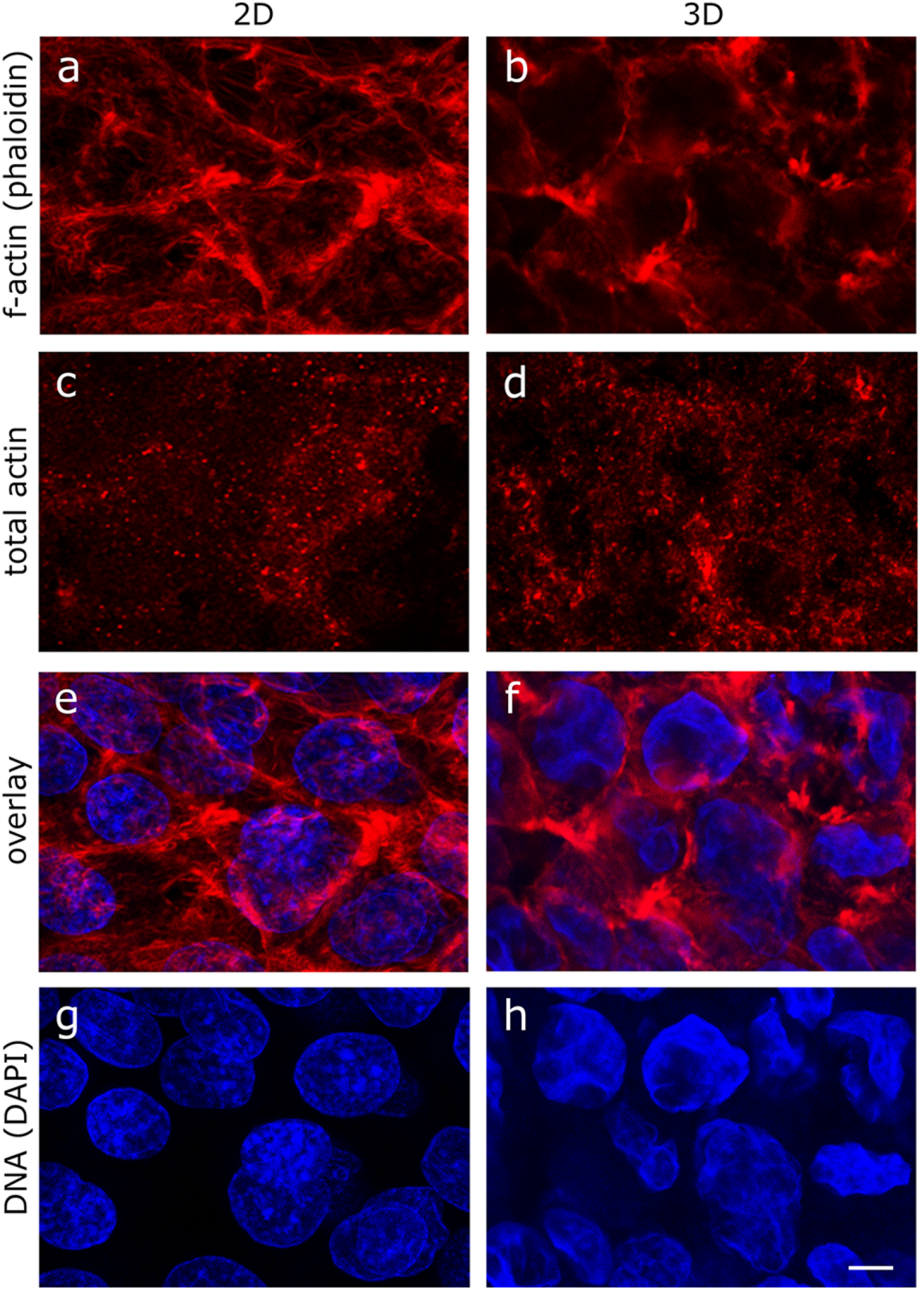
Immunohistochemical staining of actin and DAPI staining of DNA. C3A cells were grown using either the classical cell culture techniques (2D) or grown as spheroids (3D). Cells from 2D cultures were fixed directly while cells grown in 3D were fixed and sectioned. a, c, e and g: HepG2/C3A exponentially growing cells (2D), b, d, f and h HepG2/C3A cells at dynamic equilibrium. a and b: phalloidin staining of filamentous actin, c and d: total actin staining; e and f same images as in a and b overlayed with DAPI staining for DNA; g and h DAPI same images as in a and b but with DAPI staining alone. All photographs were made at the same magnification: the bar in h indicates 25 µM.

The shotgun proteomics revealed that there were essentially unchanged amounts of actin in cells grown in 3D spheroids compared to classical 2D cultures (table 1). Many proteins are involved in organising and manipulating the actin filaments [28]. In our data we observed that there was a quantative increase in many of these proteins (on average of 1.99 fold) (e.g. tropomyosin, filamin, profilin, cofilin, destrin, heat shock protein HSPH1, and the Na(+)/H(+) exchange regulatory cofactor 1 (SLC9A3R1) suggesting that there would be changes in the actin architecture (see File S1, tab actin for details of all the proteins and their grouping). This could not be conclusively confirmed by fluorescent phalloidin staining of filamentous actin (F-actin) (figure 2a and b). However, immunofluorescent staining of total actin (figures 2c and d) highlighted changes in the stippled pattern seen in hepatocytes [29] [30] [31] possibly reflecting changes at the filament-membrane attachment sites (e.g. adherens junctions) to either plastic surfaces or to other cells. For reference, figures 2e and f show overlay images for F-actin (shown in 2a and b) together with the DNA localisation (DAPI staining). Figures 2g and h show the DNA localisation only.

The proteome analysis also revealed that growth in 3D induced the utilisation of particular gene products in preference to others (‘switching’). For example the amount of the myosin heavy chain MYH10 was increased (1.57) while MYH9 was unchanged (−1.08) and MYO1C, which mediates insulin directed GLUT4 vesicle tethering was reduced (−3.69) [32].

**Figure 3:**
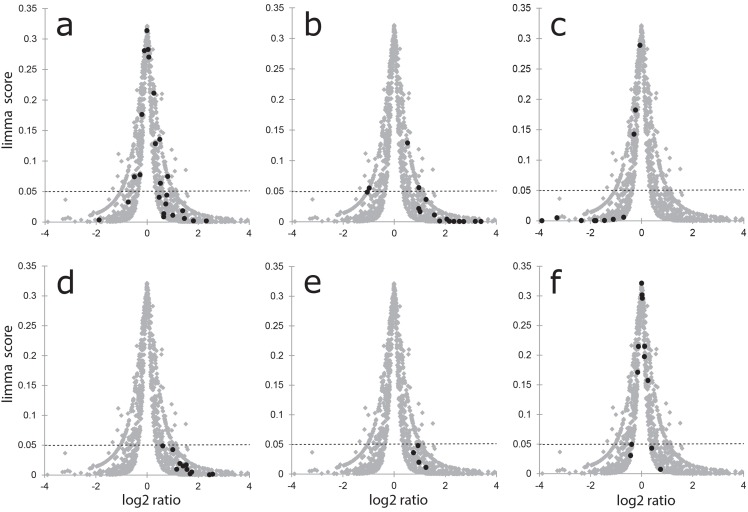
The log2 ratio change plotted against the statistical reliability (volcano plot). All proteins are shown as grey dots in the graph. Particular groups of proteins are then highlighted in black: a) Actin and actin-associated structural proteins; b) Tubulin and tubulin-associated structural proteins; c) Keratins; d) Glycolytic enzymes; e) Pentose phosphate pathway enzymes; f) TCA cycle enzymes.

A volcano plot of the log2 ratio change plotted against the minimum of limma test and rank products (limma test – see File S1, tab P1_data) for these actin and actin associated proteins shows that they are found predominantly in the upregulated segment (figure 3a). The stippled line at 0.05 on the limma test score axis indicates the false discovery rate threshold.

### Microtubules

There was a substantial increase in the amounts of the microtubule proteins (average 7.36), clearly seen by their grouping in a volcano plot (figure 3b). Dynein, which transports cellular cargo along microtubules (towards the negative end of microtubules), kinesins that move cargo in the opposite direction and microtubular core proteins were all upregulated underlining the increased importance of microtubules in 3D structures (3.20).

**Figure 4:**
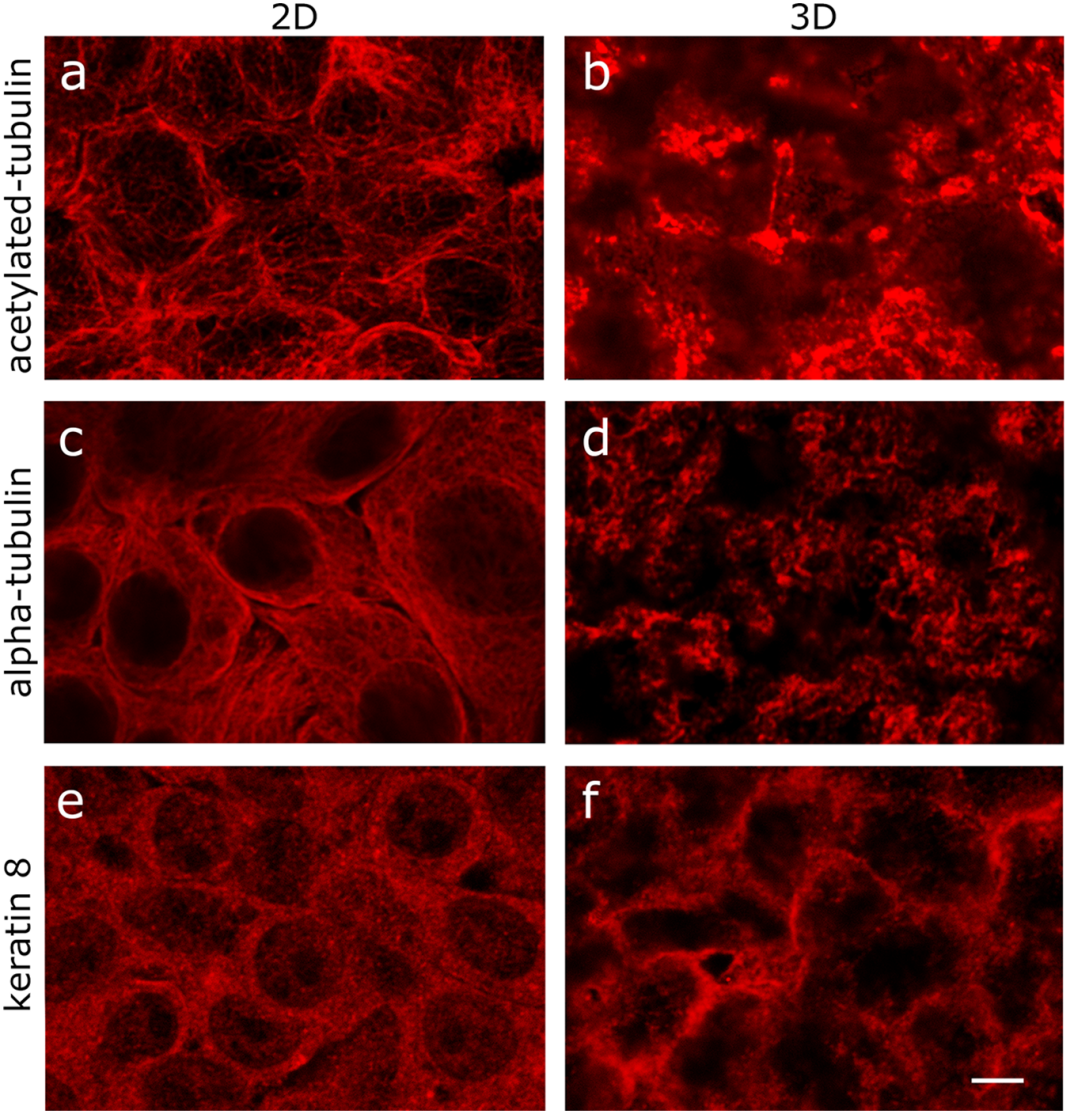
Immunohistochemical staining of tubulin and keratin. C3A cells were grown using either classical 2D flat culture techniques or as 3D microgravity spheroid techniques. Cells from 2D cultures were fixed directly while cells grown in 3D were fixed and sectioned. a, c, e and g: HepG2/C3A exponentially growing cells (2D), b, d, f and h HepG2/C3A cells at dynamic equilibrium. a and b: acetylated tubulin to highlight filaments, c and d: staining of α-tubulin; e and f keratin 8. All photographs were made at the same magnification: the bar in h indicates 25 µM.

Immunofluorescence could again confirm significant changes in cellular architecture. In hepatocytes *in* *vitro* microtubules are usually seen as exquisite thread-like patterns whereas *in* *vivo* these are replaced by a dispersed distribution in the cytoplasm [31], similar to the differences seen in figures 4a and b (acetylated tubulin) and 4c and d (alpha tubulin).

### Intermediate filaments

Several members of the third cytoskeletal class, the intermediate filaments also showed striking differences. There were marked reductions in abundance of keratins associated with simple or stratified epithelia (keratins 8, 15, 18, 19 and 23; average −6.20), (see the volcano plot in figure 3c). Dramatic increases in intermediate filaments 8 and 18 have been suggested as diagnostic markers for neoplastic lesions [33] [34]. This would suggest that the significant decrease in their expression seen here indicates that the HepG2/C3A cells, when grown in 3D, express a less ‘tumourigenic’ phenotype than when they are grown in 2D cultures. *In* *vivo*, keratin 8 is localised predominantly at the surface membrane whereas *in* *vitro*, it is localised throughout the cytoplasm (figures 4g and h) similar to that reported by Marceau for *in* *situ* and *in* *vitro* staining [31].

### The extracellular matrix and cell-cell interactions

Adherens junctions and focal adhesion plaques (both actin binding) appear to be important because catenin, integrin, alpha-actinin and fibronectin were essentially unchanged (−1.17) despite the large increase in the cell volume to surface area ratio. In contrast, the intermediate filament binding desmosomes and hemidesmosomes were reduced (−1.81) in agreement with the reduction in the amounts of intermediate filaments.

### Glucose metabolism, pyruvate dehydrogenase and the TCA cycle

In comparison to exponentially growing cells, cells in 3D spheroids are very active. The abundance of the enzymes involved in glucose, glycogen, and NADPH-pentose sugar metabolism are all strongly increased (for glycolysis by 2.92; glycogenesis 6.59; glycogenolysis 4.06 and pentose shunt 1.97) (see figures 3d and e) and summarised in table 1 [35]. These increases in glycolysis and pentose phosphate pathways extended only to the mitochondrial pyruvate dehydrogenase complex that provides the primary link between glycolysis and the tricarboxylic acid (TCA) cycle. (1.522). Thus it appears that the increase in glucose metabolism is needed to provide building blocks for the secondary metabolism of the cell.

The abundance of the enzymes in the ATP-producing TCA cycle was not increased (figure 3f) perhaps as a consequence of the high ATP levels in the spheroids.

### Oxidative phosphorylation and ATP synthase

**Figure 5:**
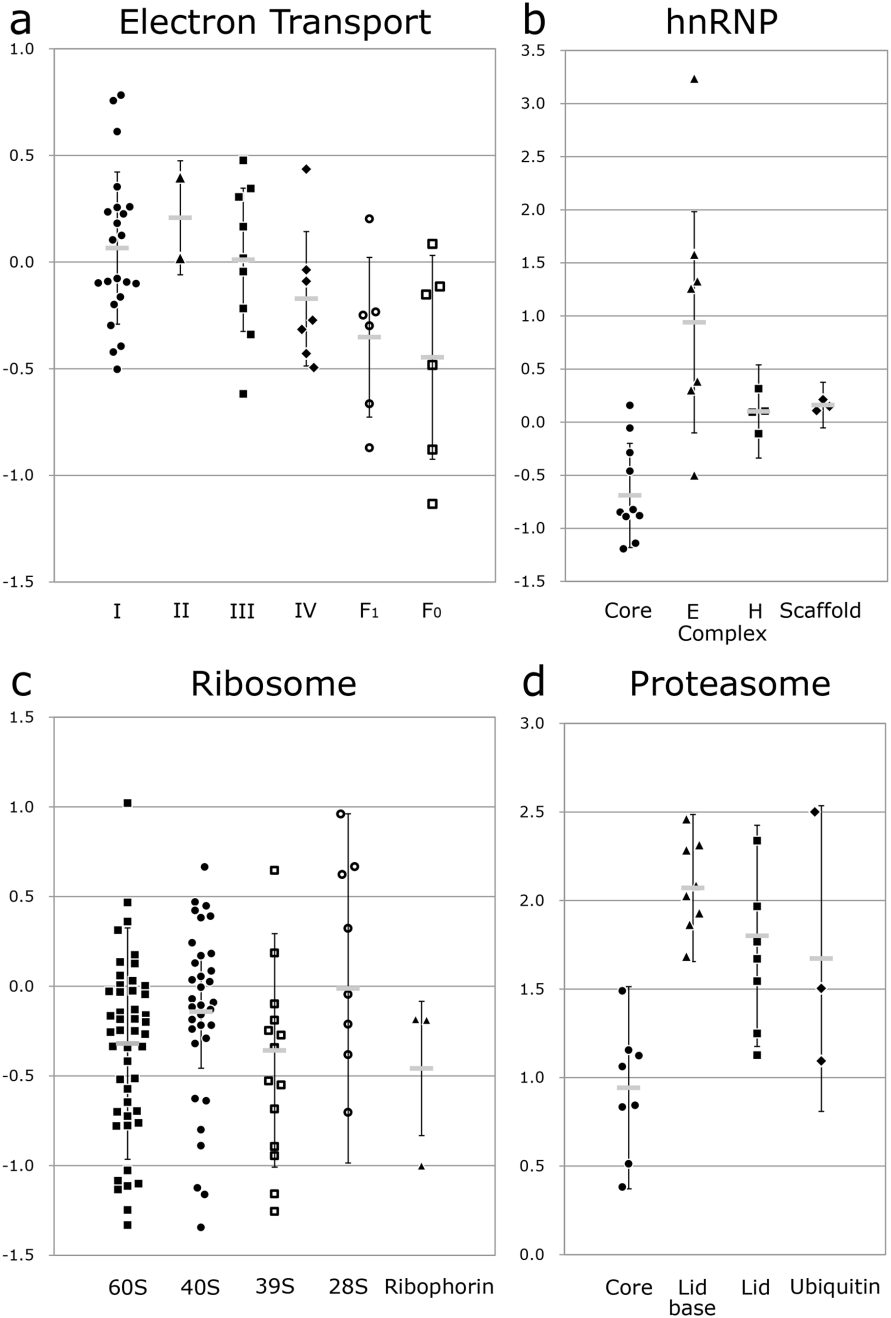
Dot plot of the ratios of protein abundance (dynamic equilibrium/exponential growth states) in various sub-cellular organelles. a) Electron transport chain: complexes I, II, III and IV and the ATPase F1 and F0. b) HnRNP: core, E complex, H complex and scaffold. c) Ribosome: cytoplasmic large 60S and small 40S subunits, mitochondrial large 39S and small 28S subunits, ribophorin linker. d) Proteasome: core, lid-base, lid and ubiquitin proteins. Error bars indicate the standard deviation of the proteins in each group and the thick grey bar indicates the average. (n = 4). For a dot blot of the spliceosome, see File S1, tab P4_Dot-plot_Spliceososme.

Corresponding to the unchanged levels of the TCA cycle enzymes, proteins in the first three complexes of the respiratory chain (I, II and III) were also unchanged. Interestingly the amounts of complex IV and both the F_o_ and F_1_ subunits of the ATP synthase were reduced by about 20% (t-test p<0.005 compared to either complex I or II) figure 5a (all possible comparisons by Student’s t-test are given in File S1, tab P4_Dot-plot_Spliceososme). Compensatory regulation of complex IV for partial ATPase deficiency has been reported before [36] so it appears that there is coordinated expression. The slight decrease in ATP synthase appears to occur despite (or perhaps as a result of) the increase in ATP levels associated with hepatocyte spheroid maturation [2] [37] and cellular polarisation [38].

### Fatty acid metabolism

Fatty acid synthesis is more active in the spheroids than in exponentially growing cells, fuelled presumably by the increased amounts of acetyl-CoA from glycolysis and NADPH from the pentose phosphate pathway and driven by feedback systems for the production of bile acids. This is best illustrated by acetyl-CoA acyltransferase which catalyzes the first step of the fatty acid beta synthesis spiral. In the peroxisomes, which are normally associated with fatty acid synthesis, acetyl-CoA acyltransferase is strongly increased (ACAA1 7.66) while in the mitochondria, which are normally associated with fatty acid oxidation, this enzyme is reduced (ACAA2-1.44).

### Cholesterol synthesis

In previous studies, we have shown that cholesterol production increased during the transition from exponential growth to dynamic equilibrium by a factor of about 10 and then stabilised at physiological levels [2]. In this study we observed that there is an increase in the enzymes involved in cholesterol synthesis (by 1.37) and that there is a strong increase (by 9.71) in the enzymes involved in the subsequent metabolism and transport of cholesterol derived products (e.g. bile acids).

### Urea production

In agreement with other studies [39] and [40] which showed that the urea cycle was defective because of gene deletion in the HepG2 cell line, the only enzyme associated with the urea cycle that was detected in these studies was carbamoyl phosphate synthetase 1 from the alternative pathway. CPS1 was increased by a modest factor of 1.50 (t-test p<0.05) and this increase must therefore be responsible for the observed increased urea production in 3D spheroids to physiological rates [2].

### Oxygen levels

**Figure 6:**
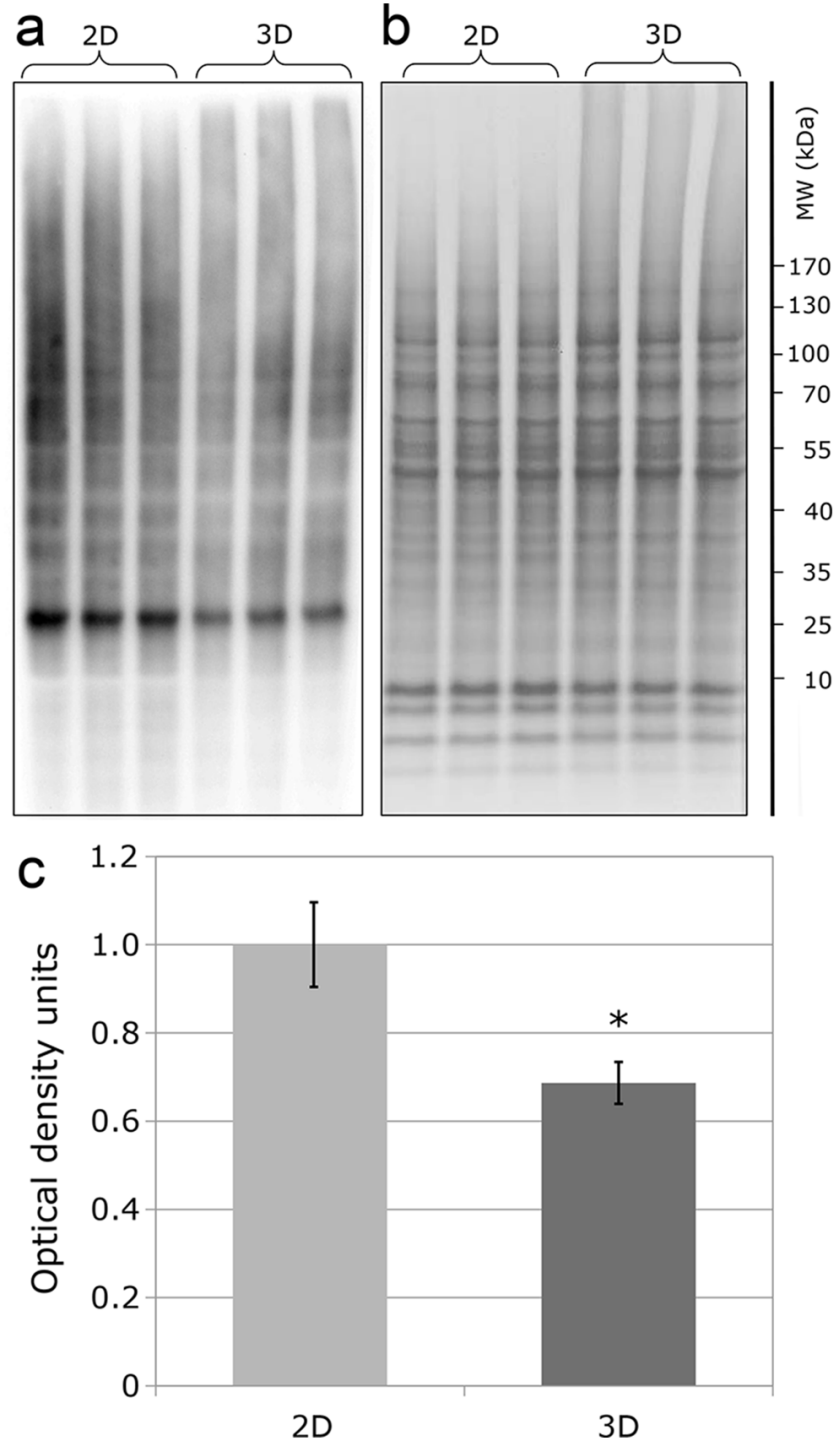
Determination of the degree of protein oxidation (carbonylation). Proteins were extracted from cells grown using either classical 2D flat culture techniques or as 3D microgravity spheroid techniques. a) OxyBlot detection of carbonylated proteins; b) loading control visualised with Coomassie Brilliant Blue total protein stain; c) Levels of protein carbonylation expressed as average % optical density: *indicates statistically significant difference (t-test, p<0.05, n = 3).

The multicellular structure of spheroids raises the question as to whether oxygen can penetrate the whole of the structure. It appears that the cells have solved this problem of oxygen transport throughout the spheroids by using a non-erythrocyte haemoglobin, which has previously been shown to be expressed in several cell types including hepatocytes [41]. The haemoglobin alpha chain is the protein showing the largest increase in the whole dataset (HBA 39.57 fold increase) and the δ chain was the third largest increase (HBD 21.06). The low oxygen tension present in the spheroids is the probable explanation for the observed fall in protein oxidation (by −1.5) as measured by the OxyBlots (figure 6).

### Cell growth

The amounts of numerous proteins, including MKI67, MCM3, MCM5, FUBP1 FUBP3 and the protein phosphatase 2 complex indicate that the majority of cells are either arrested **G_1_** or have entered **G_o_** [42]. MTOR levels suggest that this arrest is not induced by insulin, growth factor receptors or nutrients.

In support of this, we found that exponentially growing cells (in 2D culture) have more DNA per milligram protein than cells at dynamic equilibrium in the 3D spheroids, 16.47±5.87 (n = 3) compared to 5.68±1.72 (n = 9) µg DNA per mg total cellular protein (p<0.0005). There was no indication that either apoptosis (observed levels of cytochrome C and calmodulin 1 were decreased [43]) or necrosis was occurring (ATP levels were high [44] (as described previously [1]). This is in full agreement with previous observations that the doubling time of HepG2/C3A cells in exponential growth is 3 days but this falls drastically in spheroids to 17 days in 21-day old spheroids and 60 days in 42-day old spheroids [2].

### The nucleus, DNA repair and packing

Despite the fact that the cells in spheroids contain more protein relative to their DNA content, they exhibit significant increased abundance of DNA repair system enzymes (3.30), ubiquitin flagging of DNA double stranded breaks (3.47) and enzymes maintaining genome integrity (the MMS19 enzyme is increased by 14.35 fold). These changes are not indicative of apoptosis or necrosis because TUNEL staining (not shown), which detects DNA fragmentation, detected that there are very few apoptotic cells (<3%) in the spheroids. The proteomes also show histone switching, changes in epigenetic modifications and their nuclei have also undergone significant structural changes, as reflected in significant changes in the DAPI staining pattern which changes from a granular (euchromatic) to a diffuse cloudy (heterochromatic) nuclear pattern (figure 2g and h). Tvardovskiy *et al.* (personal communication) have shown that the tails of histones H2B and H3 are clipped and modified in a manner associated with selective gene transcription in the 3D spheroids of hepatocytes, but this is undetectable in 2D cultures of the same cell line (submitted).

### Oncogenes and transcription factors

Oncogenes and transcription factors present a variety of changes that do not resolve into a simple pattern of regulation. The N-myc downstream-regulated gene 1 protein (NDRG1) was present in 18.8 fold higher amounts in spheroids. NDRG1 has been shown to be a metastatic suppressor for several human cancers and has been implicated in several physiological processes including cellular differentiation and the G_0_/G_1_ arrest [45]. Similarly, the ras GTPase-activating-like protein IQGAP2 was strongly increased (5.53). IQGAP2 interacts with the cytoskeleton, cell adhesion molecules, and several signaling molecules to regulate cell morphology and has been suggested to be a tumour suppressor in hepatocellular carcinomas [46]. In contrast, the nuclear ubiquitous casein and cyclin-dependent kinase substrate 1 was highly increased (NUCKS1 12.82). NUCKS1 protein may modulate chromatin structure and it may be responsible for some of the changes noted above. High levels have been associated with various human cancers [47].

### Transcription

The change from exponential growth to dynamic equilibrium significantly modulates a substantial number of specific factors involved in the selection or repression of particular genes. For example the endothelial differentiation-related factor 1 (EDF1) was found to be reduced (−3.45). In HUVEC cells, EDF1 is reduced in quiescent cells compared to exponentially growing cells and knock-down of this gene has been shown to cause the inhibition of proliferation and the promotion of multicellular structures [48] paralleling the observations here. Another example is the increase in elongation factor 1A1 (eEF1A1 4.39) which has been suggested to be involved in the epithelial-mesenchymal transition, preventing the reversion to an embryonic-like state which was necessary for the invasive phase of metastasis [49].

### RNA processing

Numerous modulations are also seen in RNA processing. One of the families of proteins that was increased most significantly were the acidic leucine-rich nuclear phosphoprotein 32 (average 8.89). This protein family is essential for normal tissue development [50]. These proteins play an important role in stabilizing short-lived mRNAs (containing the AU-rich elements which are commonly found in proto-oncogenes, nuclear transcription factors, and cytokines). One member, ANP32A can, in a complex with SET, remodel chromatin and thus regulate transcription [51]. The oncoprotein SET plays many roles, being a potent inhibitor of protein phosphatase 2A, a histone methyltransferase enzyme and an acetyltransferase inhibitor [52]. In these studies SET was found to be increased by 2.18 fold.

### hnRNP

Heterogeneous nuclear ribonuclear proteins (HnRNPs) have distinct nucleic acid binding properties. Expression differences were observed in the various family members, which indicate that they will induce differential mRNA processing (see File S1, tab P5_Subunit_Statistics). Interestingly the core proteins of the hnRNPs themselves were reduced (average −1.61) while the hnRNP E complex (which binds poly(rC) and is a crucial modulator of mRNA stability and translation [53]) was increased (average 2.12). The H complex (which binds preferentially to guanosine-rich sequences and recognises many intronic splicing enhancers [54]), was unchanged as were the proteins involved in hnRNP-nuclear scaffold attachment (figure 5b).

### Spliceosome

The functions of most spliceosomal proteins are executed through their association or interaction with the spliceosomal RNAs or the substrate pre-messenger RNAs [55]. Dividing the detected spliceosome proteins into substructures according to Hegele and colleagues [56] demonstrates that while most of these substructures are reduced in amounts, the degree of reduction varies among the subunits (U1 -2.02; 3B -1.98; U2 -1.53; 3A -1.50; PRP19 -1.50, U2 auxiliary factor -1.15; U5 -1.20; and U4/U6.U5 complex -1.28). The exception was the DEAD box RNA helicases associated with U2 subunit and the A complex which were present in unchanged amounts (U2 1.06 and A complex 1.134, see File S1, tab P4_Dot-plot_Spliceososme and statistics in tab P5_Subunit_Statistics). All of the splicing factors detected (whether from the classical or the alternative splicing pathway) were present in reduced amounts (−1.87 and −1.39 respectively). The exon junction complex (EJC, which is important during the second phase of splicing) and components of another RNA editing complex (APOBEC, which converts cytosine to uracil) were also reduced (−1.75 and −1.37 respectivley).

However, there were exceptions to this general decrease in RNA processing enzymes. The RNA-specific adenosine deaminase which destabilizes double stranded RNA through conversion of adenosine to inosine and has been shown to play a critical role in early human development [57] was present at significantly increased amounts (ADAR 5.76) and two polyA binding proteins (which assist in the circularisation of the mRNA by binding to the eIF4 complex to promote rapid efficient transcription) were present at unchanged amounts (1.11, presumably in order to match the amounts of the eIF4 complex).

### tRNA charging

Fourteen of the sixteen tRNA synthetases detected were strongly up regulated (by 2.7). This could be correlated with either their role in translation or their recently observed critical non-translational roles in cellular homeostasis [58]. Interestingly, the 7 tRNA synthases involved in the multi-tRNA synthetase complex (MSC), which performs these additional homeostatic roles show very similar increases and have a reasonably small standard deviation (average 2.82±1.18) while those not involved in the MSC complex have a similar increase but with a large variation (average 2.53±1.96) demonstrating that the MSC members have expression that is tightly coordinated (see File S1, tab P6_tRNA-MSC).

The two mitochondrial tRNA synthases detected were also increased (1.64).

### Translation

Enzymes involved in mRNA translation show a similar trend to that seen with hnRNP and the spliceosome, i.e. that while the total level of transcription complexes appears to be increased, certain subunits and cofactors are present in decreased amounts (eIF2 -1.38; Complex 5 2.36; eIF3 core 1.47; eIF3 RNA recognition 1.15; eIF3 MPN -1.29; eIF4 1.94; PABP -1.02. For example Complex 4 (eIF4, which is the overall rate limiting complex for translation, and is involved in scanning and start codon selection) showed a clear increase in amounts while other complexes which are not rate limiting were reduced (e.g. the elongation factor 2, eIF2).

### The ribosome

The ribosome is another example, similar to the electron transport chain and spliceosome described above, where the gross stoichiometry of subunits differs from what would be expected. While proteins of the small 40S subunit is present in unchanged amounts (average −1.05), proteins of the large 60S subunit appears to be present in smaller but statistically significantly less amounts (average −1.27). Interestingly, although fewer mitochondrial ribosomal proteins were detected, they showed the same statistically significant tendency (small 27S subunit average 1.11, large 39S subunit average −1.37, figure 5C (statistical comparison data are given in File S1, tab P5_Subunit_Statistics). Whether this difference in amount can be related to the nucleolar export rate differences remains to be determined [59]. Other proteins having important functions at the ribosome were increased: for example the insulin-stimulated ribosomal protein S6 kinase alpha-3 (implicated in controlling cell growth and differentiation) was strongly increased (RPS6KA3 5.54). This will promote higher tissue like organization of the spheroids in comparison with cells grown in 2D conditions. Conversely, premature termination mutations in RPS6KA3, (and hence reductions in its amount), have been associated with the development of hepatocellular carcinomas [60].

### Protein folding

Proteins of the heat shock chaperone superfamily involved in protein folding were regulated according to their cellular localisation. For example the cytoplasmic and nuclear HSP70’s were both increased (by 2.64 and 2.61) while the HSP70’s specific for the endoplasmic reticulum and mitochondria were essentially unchanged. The peptidyl-prolyl cis-trans isomerases (PPIases) family of enzymes (which work together with the HSP70s) showed variations in expression pattern echoing their HSP70 counterpart. HSP 40, (a cofactor which accelerates HSP 70) was also increased (3.27).

All detected HSP90s were increased (average 2.51) as was the STIP1 protein that coordinates the actions of HSP70, HSP90 and the PPIases. High expression levels (as seen here) of the ATP independent chaperone HSP 27 have been shown to be inversely correlated to cell proliferation. HSP70/90/27 and PPIases are all known to promote differentiation and functionality at the expense of apoptosis [61]. The remaining chaperone systems: the ATP-driven type I chaperonin (HSP 60 and HSP10) and the ATP-driven type II chaperonin (TCP-1 Ring Complex) proteins were unchanged.

### Transport

Once proteins are translated and folded they need to be transported to various locations in the cell. Karyopherins, which transport proteins with a nuclear location sequence to the nuclear pore complex, were increased. Together with an increased amount of proteins of the RAN cycle this should increase nuclear protein import (average 3.71). Export function is also increased (average 10.32). Interestingly one of the export proteins (XPO5) is known to interact with slicer (Ago-2) in RNAi processing. The strongly increased amount of XPO5 (16.59) would thus be expected to protect against RNAi induced cytotoxicity [62].

Vesicle transport systems (endosomes, non-clathrin and clathrin coated vesicles) show an interesting diversification (summarised in table 1). Protein transport mediated by non-clathrin-coated vesicular coat proteins (COPs) can occur in both directions. Anterograde transport from the endoplasmic reticulum to the cis end of the Golgi (COPII vesicles) is unchanged while retrograde protein transport (COPI) is strongly increased (3.54). Finally, clathrin coated vesicles proteins which facilitate trafficking at the cell membrane, through the trans-Golgi network or endosomal compartments are unchanged. Proteins involved in endosomal recycling between the endoplasmic reticulum, the Golgi and lysosomes are reduced.

### Ubiquitination and protein degradation

Once a protein is tagged with polyubiquitin, it can be captured by the proteasome for destruction. Interestingly, two of the four alternative deubiquitinating enzymes detected which allow the entry of the degradation-marked protein into the proteasome, are reduced while the other two are increased. This switching once again illustrates functional specialisation. The two down regulated enzymes are USP10 (a regulator of the tumour suppressor p53 [63]) and USP39 (an indirect regulator of the spindle assembly checkpoint in mitosis [64] open circles in figure 5d). The two which are increased (roughly in proportion to the proteasome) are UCHL5 and USP7 (open squares in figure 5D). Both regulate numerous proteins broadly characterized as tumour suppressors, DNA repair enzymes, and epigenetic modulators [65] and [66].

All three parts of the proteasome (core, lid base and lid) were significantly increased. As for the ribosome and spliceosome, these increases were not stoichiometric relative to each other but were present in different amounts (figure 5D), (p<0.0005, see File S1, tab P5_Subunit_Statistics).

## Discussion

This study has revealed that there are changes throughout the entire cell as the cell adapts from exponential growth to a dynamic equilibrium. During this process, the cell establishes a highly efficient infrastructure allowing it to execute advanced physiological functions characteristic of tissues.

The observation that the amounts of one of the protein subunits of a heteromer are highly correlated to the other protein subunit demonstrates very clearly that the cell regulates protein expression of every protein very accurately both during exponential growth and at metabolic equilibrium. This observation is strongly supported by protein expression levels within other protein substructures in other multiprotein complexes or in most of the protein functional groups described above (where the exact stoichiometry is more difficult to define). These ‘groups’ include structural proteins (tubulins and keratins), nucleosome (H2A+H2B; H3+H4), ribosome (large and small subunits, both cytoplasmic and mitochondrial), spliceosome substructures, proteasome (core; lid-base and lid), MSC complex, electron transport chain complexes (I; II, III and IV) and ATP synthase (F_0_ and F_1_). This regulation also applies to proteins that are functionally related, for example in glycolysis, the TCA cycle or the pentose phosphate pathway (where the standard deviation of expression for enzymes in these pathways was ±0.49, 0.26 and 0.15 respectively). This accurate regulation of protein expression levels would not be necessary if it did not bear some relationship to their functional activity level in the cell.

Superimposed on this accurate regulation of proteins within structural or functional groups, was the observation that different subcomponents of macromolecular complexes, when considered as a whole, display subtle but statistically significant changes in their stoichiometry (based on t-tests (see File S1, tab P5_Subunit_Statistics). The large subunit of the ribosome (both cytoplasmic and mitochondrial) for example is reduced in its ratio compared to the small subunit. The subunits of the electron transport chain I, II and III are unchanged while both subunits of V (the F_o_ and F_1_ ATPase) are reduced. In the proteasome the lid is increased to the highest extent compared to the lid-base and the core. It appears that complexes designed to initiate processes (e.g. the transcription initiation complex, the hNRNP E complex, the 40S and 29S ribosomal small subunits, and the proteasome lid) are typically present in higher amounts than their corresponding ‘executive’ subunits. Presumably the purpose of this is to capture the ‘substrate’ (mRNA, or protein) effectively so that the executive subunit can be kept busy and increase cellular efficiency. The spliceosome U1 subunit (which captures the hnRNP) is however reduced and thus appears to be an exception to this hypothesis.

The changes in enzymes involved in cholesterol and urea production are modest (1.37 and 1.50 respectively), but must be sufficient to increase cholesterol and urea by factors of 10 and 3 respectively to reach physiologically equivalent *in* *vivo* levels [2]. The fact that significant physiological changes can be achieved by subtle changes in protein expression is in excellent agreement with the need to precisely control the amounts of the various proteins in the cell. Furthermore, it suggests that cellular architecture, which was found to be significantly modified, may play an important role in the efficient metabolism of the cell. Thus, the attainment of *in* *vivo* physiology and *in* *vivo* intracellular architecture may go hand in hand.

Many of the proteins characterised in this study have also been reported by others as associated with cellular differentiation, growth potential, the loss of immortality, increases in tumourigenic or metastatic potential. In almost all cases the expression changes reported here (both above and in File S1) correspond to higher differentiation, lower growth potential or tumourigenicity. In our opinion, these characterise normal tissue *in* *vivo* and illustrate that 3D spheroids are an attractive surrogate model for studies of human tissue.

Although we have referred to the cells being at equilibrium, they are actually only close to equilibrium. First of all, there are significant variations in nutrient and gas levels in the cultures because the culture medium is only refreshed each 48 h [37]. In addition to this, the cells in the spheroids are exposed to different environments: cells at the surface will have more nutrients, higher pO_2_ and lower pCO_2_ levels than internal cells. These gradients may in some way resemble gradients present in tissues and variations in the blood composition over time (e.g. after a meal). This may give rise to ‘gradients’ in the abilities of the cells, for example cells at the surface of the spheroid may have a faster cell cycle (due to better ‘nutrition’) than those deeper in the spheroid. These differences may mimic the different zones in the acini of the liver. Each acnus is usually divided up into three different zones (the periportal, intermediate and pericentral zones) and numerous publications have demonstrated that the (genetically identical) hepatocytes in these zones are metabolically heterogeneous. The hepatocytes in the periportal zone (where the oxygen and nutrient levels are highest) are more involved in gluconeogenesis and fatty acid β-oxidation, while pericentral hepatocytes are more engaged in glycolysis, xenobiotic metabolism and lipogenesis [67]. The results presented here must therefore be considered as an average picture. In other words, cells from different environments within the spheroid would be expected to express differences in their proteome. Similar considerations would be relevant to cells in tissues.

## Conclusions

**Figure 7:**
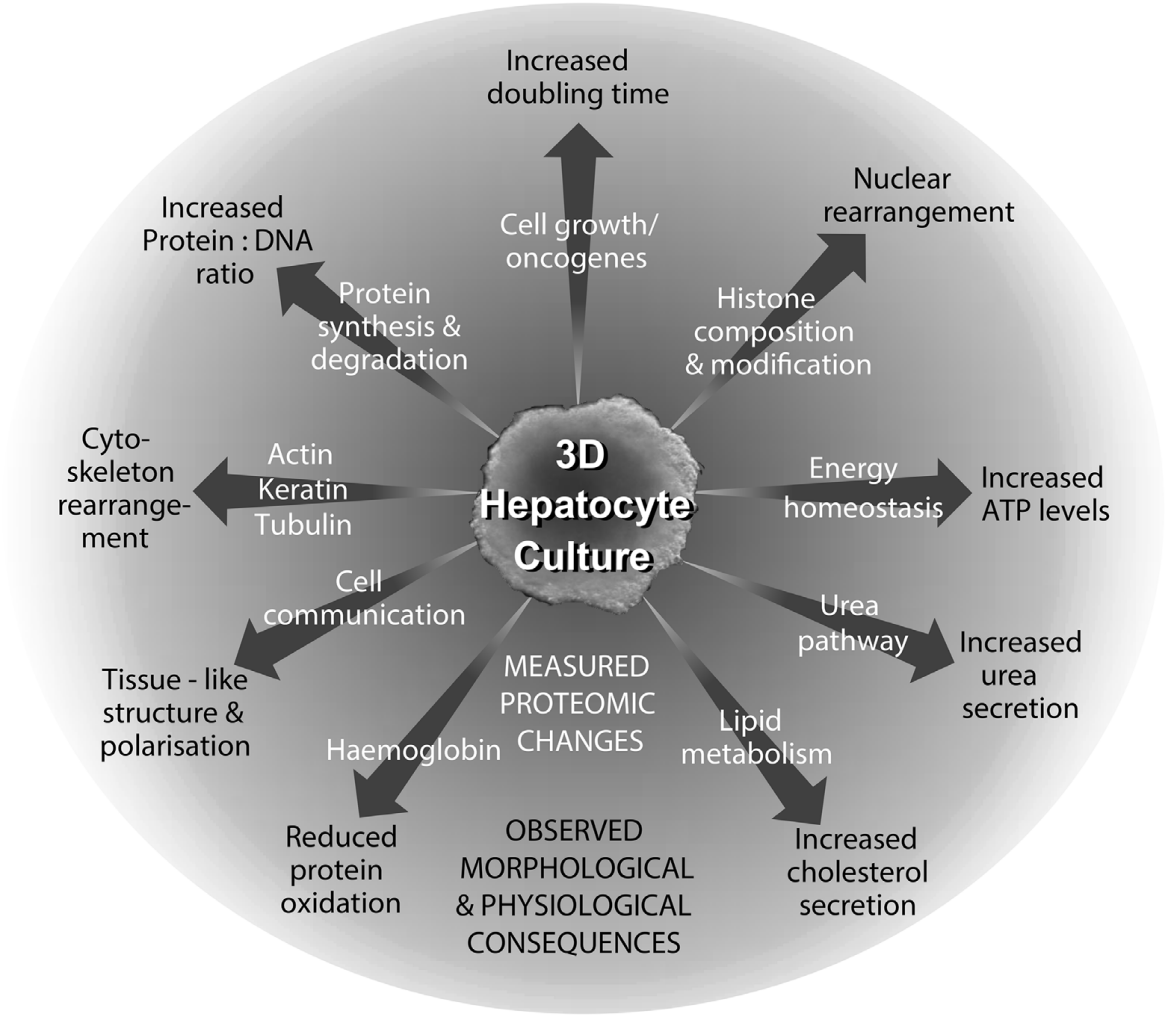
Relationship of the changes in the proteome following its adaptation from 2D to 3D culture with structural and physiological properties.

For the first time we have shown that the proteome of exponentially growing cells and cells at dynamic equilibrium are dramatically different. There are significant changes throughout the cell which affect almost every aspect of cellular metabolism. These changes are the foundation for profound differences in architecture, functionality and physiology (figure 7). Cells at dynamic equilibrium in 3D spheroid cultures are highly focussed on functionality and can mimic the performance of tissues. Exponentially growing cells in 2D cultures have sacrificed these attributes and are dedicated to replication instead. These observations have important consequences and should be taken into account when data, obtained using classical cell culture, are extrapolated to the human organism.

## Supporting Information

File S1 All the supporting information has been collected into one file for the convenience of the reader. The material is presented as an excel spreadsheet to make it easily accessible. In File S1, the information is presented on different tabs. The supporting information is divided into 6 parts: **TOC. Table of contents; P1_data. Basic proteomic data.** The basic data set; **P2_Histogram. Distribution of protein changes against the degree of change.** The ratio of change for each protein (in HepG2/C3A cells growing at dynamic equilibrium in 22 day old 3D spheroid cultures divided by that seen in exponentially growing cells grown using the classical 2Dculture) was grouped and the number of proteins in each group was then plotted against the ratio of change. The dotted line is the reciprocal plot to emphasise the skewedness of the distribution. Fold change is expressed as the log2. n = 4; **P3. Extended results description.** This section provides a detailed explanation for the statements made in the result section. It has multiple tabs corresponding to the headings used in the ‘results’ section; **P4_Dot-plot_Spliceosome. The Spliceososme.** Spliceosome proteins have been grouped according to subunit structure plotted against their ratio of change (3D/2D). Fold change is expressed as the log2. Error bars are shown and the average is indicated by the broad grey bar. n = 4; **P5_Subunit_Statistics. Statistical comparisons of protein expression in macromolecular subcomplexes.** Proteins were grouped into sub-complexes and all possible combinations of the groups were compared using a standard two-tailed T test; **P6_tRNA-MSC. Comparison of the changes in the ratio of abundance of tRNA synthetases which are part of the MSC complex with those that are not.** tRNA synthetases have been grouped according to whether they form part of the MSC complex or not and plotted against their ratio of change (3D/2D). Fold change is expressed as the log2. Error bars are shown and the average is indicated by the broad grey bar. n = 4.
(XLSX)
