# Correction: The Cultural Divide: Exponential Growth in Classical 2D and Metabolic Equilibrium in 3D Environments

**DOI:** 10.1371/journal.pone.0118050

**Published:** 2015-02-11

**Authors:** The PLOS ONE Staff

There is an error in the third sentence of the “The nucleus, DNA repair and packing” subsection of the Results. The correct sentence is: The proteomes also show histone switching, changes in epigenetic modifications and their nuclei have also undergone significant structural changes, as reflected in significant changes in the DAPI staining pattern which changes from a granular (heterochromatic) to a diffuse cloudy (euchromatic) nuclear pattern (figure 2g and h).
